# Medical Wards of the Mercy Hospital

**Published:** 1873-12-15

**Authors:** N. S. Davis


### MEDICAL WARDS OF THE MERCY HOSPITAL.

SUBSTANCE OF A CLINIC ON INTESTINAL WORMS. SERVICE OF PROF. N. S. DAVIS.

*Tænia Lata—Lumbricoides—Ascarides.*—Gentlemen: The patient before you is a middle-aged man, of foreign birth, and having the outward appearance of fair health. He came to the hospital several weeks since, on account of some surgical ailment, from which he was readily relieved, and was then transferred to the medical department, on account of some cough and unpleasant feelings in the abdomen, attributed to *tape-worm.* On inquiring into his history, it was ascertained that he had been many months troubled with variable appetite, a variety of morbid sensations in the abdomen, seldom amounting to pain, slight costiveness, disturbed sleep at night, and more or less mental despondency. During most of the time, he had passed from his bowels, every two or three days, something which he represented to be worms.

On examination, these proved to be detached pieces *tænia lata*, or tape-worm. They were white, flat pieces, varying from one-quarter to three-quarters of an inch in length, and about one-quarter of an inch in width. Sometimes several of these pieces would pass united, end to end, as I will show you in a few moments. This patient had at different times tried several remedies for the expulsion of his worm, before he came to the hospital; and during the first two weeks that he was in my hands he took pretty full doses of oil of turpentine, santonine, chloroform, and the fluid extract of male fern, but all with no apparent effect upon the worm. About three days since, however, he commenced taking drachm doses of the ethereal oil of the felix mas or fern, and repeated them before each meal and at bed-time, taking only very light food. At the end of the second day, after having taken, in all, an ounce of the medicine, he had free evacuations from the bowels, accompanied by the expulsion of a large mass of the worm—the larger portion of which I show you in this basin, fresh, as it appeared at the time of expulsion. You see around the margin several short, flat pieces, or joints, detached from the rest, such as he has been in the habit of passing every few days for many months. In the center of the vessel is this large mass, consisting of the same short, flat pieces, united in a continuous chain, coiled upon itself, but which, if it were extended into a straight line, without breaking, would measure twenty feet or more. At this place, you see it appears to end in a long, narrow and tapering point, which is probably the head of the worm. It is probable, therefore, that the whole of the animal has been expelled, and that the patient will remain free from further trouble from that source. So long as the head remains in the alimentary canal, the sections composing the body will be reproduced more or less rapidly.

The effects of the presence of a tape-worm on the general health of the patient, are not strongly marked. The patient before you is neither emaciated nor anemic. His principal suffering is from obscure morbid sensations in the abdomen, and from mental anxiety. And such has been the case with a large majority of those coming under my observation. Sometimes, however, the presence of the parasite causes more serious disturbance of the nervous system.

I have a patient now who has been suffering for two or three years from very severe neuralgic pains in his head, spine, and chest, with restlessness at night, and generally constipated bowels. At one time his physician found the pain in the back of the head and neck so severe as to create apprehensions of cerebro-spinal meningitis. He has been subjected to a great variety of treatment, addressed to the digestive and nervous systems, but without permanent relief.

Recently it was discovered that he was passing, almost daily, sections of a *tape-worm*. How far this may have been the cause of his long-continued nervous suffering remains to be proved. I advised for him full doses of the ethereal preparation of male fern, a day or two since, but have not learned the result.

About thirty years since, when practicing in the interior of New York, I was called to a young lady aged about 18 years, who had been having several paroxysms of general convulsions, resembling epileptic paroxysms. The tongue was coated, bowels costive, pulse 90 per minute, with some heat of skin, and general feeling of soreness, like muscular rheumatism.

These symptoms led me to prescribe six powders, each containing five grains of powdered colchicum root, and one grain of calomel: one to be taken every four hours. Before she had taken all the powders she began to have free intestinal evacuations, and voided a very large quantity of tape-worm. She soon recovered good health, and had no return of convulsions.

The different varieties of *tœnia*, or tape-worm, chiefly occupy the ileum, or middle portion of the alimentary canal. They have occasionally been expelled under the influence of a great variety of remedies. At the present time the profession rely more upon the efficacy of felix mas, koosso, and the pumpkin-seeds, than upon any other remedial agents. The most reliable preparation of the first of these is the ethereal oil, which may be given in doses varying from ten to sixty drops, repeated three or four times at intervals of three hours, and followed by a cathartic.

During the time of its administration the patient should either fast or take very light nourishment only. The koosso, I have never used, although it has been very highly recommended by some practitioners. During the last five years I have caused the expulsion of more tape-worms by the use of pumpkin-seeds than by any other remedies. Success in the use of this remedy, however, depends almost wholly on the *manner* of its use. Two ounces of the seeds should be thoroughly bruised or broken up and put into a pint of water boiling hot, in the evening. Stir them up several times while cooling, and let the dish stand until morning. Let the patient retire at night without any supper, and in the morning drink the whole pint of pumpkin-seed infusion, instead of breakfast. If the bowels do not move freely by one or two o'clock, P. M., let him take a tablespoonful of castor oil, mixed with a teaspoonful of oil of turpentine. After it has operated freely he may take food as usual. I have known this method to sueceed in expelling the whole mass of the worm in a goodly number of cases.

There are two other species of intestinal worms, much more common than the *tœnia*, or tape-worm.

The lumbricoides, or common round worms, are frequently met with in children, and sometimes in adults. They may be found in the stomach, or any part of the intestines, though they live mostly in the ileum, and colon. Their existence is generally indicated by fulness of the abdomen, capricious and often voracious appetite, disturbed sleep, and occasional attacks of transient fever; and yet they are often seen in the intestinal evacuations of persons who have appeared to be in previous good health. They are generally easily expelled by the judicious use of either oil of turpentine, santonine, spigelia marylandica, or carbolic acid, aided by mild laxatives. For convenience of exhibition, certainty of effect, and safety from bad consequences, the santonine is the best remedy for this species of worm. It may be given in doses of from one to five grains, according to the age of the patient, repeated every four hours, until three or four doses have been taken, and then follow it by a cathartic.

The remaining variety that I will allude to is the *ascaris*, or pin-worm. This is a small worm that inhabits the lower bowels, chiefly the rectum, burrowing in the folds of the mucous membrane, producing itching and titillation locally, and sometimes, more general nervous derangement. They are often passed in great numbers, exhibiting a lively motion, and not unfrequently crawl out of the anus, when there is no other evacuation. The locality of this species of worm in the rectum, the deposit of its larvæ in the folds of the mucous membrane, and its rapid multiplication, makes it very difficult to get entirely rid of, by any quantity of vermifuge and cathartic remedies given by the mouth. For several years past I have abandoned all such treatment for ascarides, and have substituted injections into the rectum. One tablespoonful of common table-salt, dissolved in a pint of cold water, and enough of it used as an injection to fill up the rectum pretty well, not only kills the existing worms, but also destroys their larvæ. A salt-water injection every second or third night, until three or four had been used, has uniformly and effectually relieved such cases as have come under my care.

